# Beyond Tokenism: Making Lived Experience Leadership Visible in Co‐Produced Research Authorship

**DOI:** 10.1111/hex.70665

**Published:** 2026-04-16

**Authors:** Patte Randal, Stefan Heinz

**Affiliations:** ^1^ Expertise by Lived/Living Experience; ^2^ Department of Psychological Medicine University of Auckland Auckland New Zealand; ^3^ Division of Health University of Waikato Hamilton New Zealand

**Keywords:** authorship, co‐production, epistemic injustice, epistemic justice, ethics, lived experience, porous solidarity, power

## Abstract

**Background:**

Co‐production research values the lived/living experience (LE) of people navigating health challenges. Despite this, traditional academic authorship often disregards power dynamics that participatory research seeks to address.

**Main Body:**

This critical reflection argues for centring LE collaborators as first authors in co‐production research. We analyse authorship through Foucauldian power/knowledge dynamics, Derrida's deconstructive ethics and Levinasian ethics of responsibility, drawing on Critchley's synthesis of these traditions. This multiple lens reveals a tension: co‐production research requires political solidarity (‘us’) to challenge epistemic injustice, yet demands ethical vigilance to preserve individual voices. The concept of porous solidarity is useful here. Acknowledging the value of experience‐based expertise by means of first authorship embodies this framework: it redistributes epistemic authority and shifts research from studies about populations to studies by/with those populations.

**Conclusions:**

First author placement of experience‐based experts where appropriate, plus explicit acknowledgement of all researchers' relevant LE where freely given, offers both an ethically responsive approach and a practical strategy for aligning co‐production processes with publication practices. By centring LE collaborators as first authors and acknowledging the LE of academic researchers, co‐production research teams can embody the principle rooted in disability activism, of ‘nothing about us without us—leading’ in academic literature. This supports epistemic justice, creates solidarity and builds capacity within communities conducting research.

**Patient or Public Contribution:**

This lived experience–led article was co‐produced by two researchers with lived experience. Experiential expertise shaped the conception, argument development, drafting and critical revision of the manuscript and the practical implications presented.

## 1 Introduction

### 1.1 Positionality and Standpoint

This manuscript emerges from tensions I (Stefan Heinz) have encountered while engaging in co‐design within health research, as well as our joint commitment to more authentically recognise the value of lived and living experience (LE). My participation in co‐design studies has raised recurring questions about authorship. In co‐designed studies with community members or patients (termed here ‘LE collaborators’), I witnessed a mismatch between the egalitarian aims of co‐design and the hierarchical conventions of academic authorship. Co‐design seeks to democratise knowledge production through inclusion and co‐generated research questions, methods and interpretations with people with lived experience of a health issue. Yet when it comes time to publish, authorship conventions often reintroduce hierarchy: the academically trained researcher is usually listed first, while nonacademic LE collaborators may appear later in the byline or be relegated to the acknowledgements.

Stefan invited me, Dr. Patte Randal, to collaborate in co‐authoring this reflection, as I am a doctor trained in psychiatry with lived experience of recovery from psychosis. As a postgraduate student, having candidly told my professors about my recovery from psychosis and what I had learned, and how it was informing my practice. I was told:

You are too open, too honest and have too much integrity to be a psychiatrist in.

My theoretical framework, I was told, did not fit with what was considered standard practice [1]. Subsequently, as both a clinician and researcher with lived experience, I have published quantitative research on recovery‐oriented interventions, qualitative research on the experiences of doctors who have become the patients of psychiatrists, reflections on epistemic injustice in medical training, and have experienced both inclusion and erasure in authorship practices.

Since this analysis will critically examine who holds power as a result of producing knowledge, we wish to disclose to readers what positionality and potential conscious/unconscious biases we both represent. I, Patte, am of Jewish British descent, a woman with a doctorate in psychology who worked for 30 years as a medical doctor trained in psychiatry with disclosed lived experience since 1987. The dual role initially resulted in discrimination and was devalued by the medical institutions. However, individual academic senior colleagues offered their support in the publication process.

I, Stefan, am of Swiss and German descent, a male, and hold a position in academia. This collaboration and the trust that grew through connecting with Patte created the conditions for a self‐disclosure I had not previously felt able to make. Aspects of my own lived experience shaped my professional pathway, which began with studying Information Technology and led me to embracing a career in nursing and to my doctoral research. However, the structures I work within often decide which aspect of my knowledge counts before I enter the room.

We wish to identify ourselves by our ethnicity and socioeconomic status since both these statuses impact whose knowledge we have learned to respect and whose voices we have become accustomed to listening to. We acknowledge that the unearned authority associated with each of our identities is part of the dynamics examined in the next section.

As co‐authors, we offer this paper as a reflective, philosophical and conceptual analysis in collaboration. Rather than simply reporting empirical findings, we examine authorship through our shared commitment to co‐design and the differences in our experiences as researchers with lived experience. Our collaboration created a closeness in the writing that brought into view many of the dynamics we discuss, especially the challenge of finding language precise enough to hold both shared purpose and difference. We did not treat these moments as data; instead, they deepened our understanding of authorship as ethically and relationally grounded. We interpret these insights through Foucauldian and Levinasian lenses, supplemented by Critchley's synthesis of deconstruction and ethics, which led us to the concept of porous solidarity between us, and we hope, with our readers.

### 1.2 Scope and Definitions

This argument applies specifically to co‐production and co‐design research, collaborative processes where people with lived experience are involved in co‐planning, co‐design, co‐delivery and co‐evaluation of initiatives [2], applied here to research studies. ‘Nothing about us without us’ is one of the central tenets of disability rights activism, in which disabled peoples sought to define their own existence and make decisions for themselves regarding how they live their lives [3]. We use ‘LE collaborator’ consistently throughout to denote research partners whose primary contribution derives from their experiential expertise, while acknowledging that many researchers (including ourselves) hold both academic credentials and lived experience. The key distinction is not binary but concerns which aspects of expertise are valued, whose expertise is foregrounded and how this is recognised in publication. The context around appropriate self‐disclosure of LE for those of us who dwell in both worlds is discussed.

### 1.3 The Problem: Authorship Hierarchies in Co‐Production

Although authorship on scholarly papers is one of the main forms of academic credit and voice, each discipline has its own norms of authorship that were established historically through a normative separation between researcher and subject, and between researcher and method. In many academic areas, primarily in the biomedical and health sciences, the first author is the individual who has contributed most of the work. The last author is usually the senior investigator or principal investigator who secured funding and/or supervised the research. Each of these positions ‘carries special weight’ with respect to prestige and career advancement. Other authors are ‘typically listed in decreasing order of importance’; therefore, being first author or last author represents considerable academic capital [4].

In co‐design and co‐creation practice, this ordering clearly privileges those with academic qualifications and can support their promotion within the field. The fact that for many of us, LE has slowed down or prevented academic achievement does not, of course, represent a lack of capacity. Yet even when nonacademically qualified LE contributors shape priorities that become research questions or collect crucial datasets, they may be placed in middle authorship positions or relegated to the acknowledgements section, rather than being recognised as lead author. A recent commentary on young co‐researchers in health research similarly noted that even co‐production projects with the best intentions can unknowingly ‘perpetuate hierarchical and inequitable practices’, as professional researchers fall back on the status quo, relying on traditional power dynamics that relegate nonacademic lived‐experience partners to the sidelines [5]. In other words, even in the spirit of ‘partnership’, the institutionally positioned researcher is often (implicitly) regarded as the primary knowledge producer whose name deserves prominence. This can be seen as epistemic injustice [6, 7].

The burden of conducting most of the work of research typically falls on early‐career researchers, including collecting and analysing data, often working in precarious conditions and at lower levels of compensation. Senior researchers receive the majority of the academic recognition (last author credit). Many early‐career researchers have their own undisclosed LE. Therefore, if we adopt the principle that first authorship should go to the researcher with the least entitlement, this could lead to inequitable treatment of some early‐career researchers who do much of the daily labour but have undisclosed LE, rather than promoting greater equity across the authorship team. Any reforms to authorship will need to be negotiated with the entire research team, not dictated by a set of rigid rules.

### 1.4 The Hidden Prevalence of Lived Experience

As hinted to above, there is a growing body of research showing that many academics who would be regarded in the co‐production (or other research) team as ‘non‐LE’ are, in fact, managing our own histories of mental distress, trauma, disability or marginalisation but choose not to disclose due to well‐documented risks. Studies consistently report that academics fear professional stigma, reduced credibility and negative career consequences when disclosing lived experience, particularly in mental health fields where expectations of neutrality and ‘objectivity’ remain strong [8, 9]. The developing lived experience leadership literature provides evidence of the benefits of disclosure as an area of epistemic authority and relational trust, but also demonstrates risks to individuals, particularly to those in early career or precarious positions [10].

Surveys of university staff indicate high rates of undisclosed mental health challenges, with disclosure rates far lower than prevalence estimates would predict, suggesting that silence is often a rational response to institutional cultures that penalise vulnerability [11]. This creates the appearance of a clean LE/non‐LE divide, when empirically the distinction reflects patterns of concealment shaped by structural stigma rather than actual differences in experience. Recognising this dynamic complicates simplistic authorship categories and underscores the need for authorship practices that account for the full spectrum of experiential knowledge, including that which remains intentionally or necessarily unspoken.

If LE‐first authorship becomes the standard form of authorship, there may exist unspoken pressures upon those with dual identities (i.e., researcher/LE) to disclose their identity before they can feel comfortable doing so. An early career researcher, who has used their experiential knowledge to develop a project, yet has not made this known, will face a difficult decision: to claim credit for the contributions they have made, thereby risking professional consequences; or to remain silent and thereby miss out on the benefits of the reforms which seek to recognise their LE. This is particularly true for researchers who identify as both LE and have one or more additional marginalised identities (e.g., marginalised ethnicity, disability or socioeconomic disadvantage, etc.). These individuals already experience higher levels of vulnerability, and disclosing their LE status can further exacerbate this. Therefore, authorship practices should include supportive approaches such as early discussions at the team level regarding how the LE status will be recognised. Policies could state that nondisclosure will not result in penalties and disclosure will be valued.

Furthermore, a survey of researchers and ‘experts by experience’ found that most nonacademic LE collaborators (63.9%) and seemingly non‐LE academic researchers (70%) agreed that an author's LE ought to be acknowledged publicly in publications [12]. Many suggested that an author's affiliation or byline include their LE role. Nevertheless, the same survey indicated that, in practice, only 65% of LE co‐authors reported their LE was always or sometimes acknowledged, and 15% said it was never recognised. Miles et al. [13] highlighted the same gap, noting that authorship guidelines rarely provide guidance on how to identify and/or acknowledge LE academic co‐researchers or nonacademic LE collaborators in co‐produced research.

Some may argue that authorship should reflect actual contribution to writing and analysis; that our proposal prioritises politics over meritocracy. We respond that ‘contribution’ itself is a contested concept shaped by existing power structures. Who holds the moral authority? If an LE collaborator's insight fundamentally shaped the research question, informed data interpretation, and ensured findings resonate with affected communities, why should current facility with academic prose determine authorship order? The key argument is that perspective should inform authorship placement rather than level of seniority or academic skill alone. This is not about charity; it is about recognising that perspective and lived expertise are as valuable as technical skills.

This misalignment between the ideals of co‐design/co‐authorship and our publication practices suggests the need for cultural change. If we take seriously the principle of ‘nothing about us without us’, a principle whose power, as Charlton [3] showed, derives from the fact that it was articulated by the communities it describes, we should ensure that LE expertise is not only present in the research process, but also clearly recognised in authorship, so that the people whose lives and communities are most affected are visibly authorised to speak in the academic record.

### 1.5 Working Within Existing Guidelines

Crucially, nothing in the ICMJE guidelines prohibits a nonacademic LE collaborator from being the first author. The ICMJE requires that authors meet all of the following criteria: substantial contributions to the conception, design or data interpretation; drafting or critically revising the work; final approval; and accountability for all aspects. On its face, nothing prevents nonacademic LE collaborators from being authors; in fact, many collaborators substantially influence the conception of the work and interpret the data through their lens of LE (meeting criterion 1), and they regularly review and provide comments on drafts (criterion 2).

The ICMJE [4] specifically states that the order of authorship should be a joint decision of all co‐authors, and that co‐authors may need to justify the order. This allows a team to decide that the person whose LE shaped the study should be the first author, since this shaped the conception and understanding of the research. In clinical research, the last author position typically signifies the senior researcher or mentor. Thus, centring the LE collaborator first and the institutionally positioned researcher last represents an acceptable model within existing conventions: the person whose lived experience grounds the work is acknowledged first, while the person who provides oversight and takes institutional responsibility for the work is last. In some instances, this may even be the same person, but their LE contribution would rarely be prioritised or acknowledged. It is possible to upturn the typical hierarchy within existing norms; it is more a matter of disposition and mindset than breaking the rules.

Journal editors are increasingly receptive to some of these changes. A survey of 112 medical journal editors found most supported patient co‐authorship in principle, though few journals had formal policies to guide implementation [14]. Journals like Research Involvement and Engagement regularly publish articles with patient co‐authors and reviewers. More significantly, BMJ now requires Patient and Public Involvement (PPI) statements in all research articles, reporting on how patients or the public contributed to the study design, conduct and dissemination [15]. By offering writing support through mentoring or pairing, teams can enable a less academically experienced person to fulfil writing‐related requirements for authorship. In some cases, nonacademic LE collaborators do want to be authors and have their expertise called as such, and empowering them could be as simple as saying, ‘We would like you to take the lead role in writing the paper. How can we support you to do so?’

What is less clear is how we go about making it safe for LE academics to disclose our LE expertise where relevant and receive public recognition for this aspect of our contribution. This is beginning to happen, but we still have a long way to go (citation removed for peer review). Hopefully, this manuscript will help.

### 1.6 Authorship and Power: A Foucauldian Analysis

Foucault argued that knowledge is not autonomous of power; power and knowledge directly imply one another [16]. In Foucault's analysis, what society (or an institution such as science) accepts as ‘truth’ is contingent upon the balance of power, with the powerful often determining whose knowledge gets recognised [17]. Academic authorship is a clear example of this power/knowledge relationship. The position of the author is not just an insignificant technicality; it is a position within the discourse of science that carries authority. In Foucault's terms, the ‘author‐function’ helps to establish the legitimacy of knowledge claims made in a text. When a scientific paper bears one's name, especially as first author, it signals that one is recognised as a creator of knowledge: someone authorised to interpret data, take a position and draw conclusions.

This relationship can discount experiential knowledge by privileging forms of knowing that are filtered through academia and treated as ‘real’. Foucault's notion of discourse would encourage us to ask: who is speaking in the text, and how does the institution of authorship maintain and legitimise existing hierarchies of knowledge? If we create an academic culture in which we agree to reorder authorship so that LE is first, we can disrupt the hierarchy of discourse. By creating a new author‐function, authors challenge readers (and other researchers) to see the person with LE as a legitimate knowledge producer. This could transform the power relations embedded in the institution of knowledge. The metaphorical ‘voice’ of the publication is reshaped when the first author is a person speaking from lived experience of the issue.

However, we must ask: could suggesting experience‐based first authorship as a form of ‘solution’ become what Foucault might have called a new disciplinary mechanism? While we are promoting a specific practice, might we also be creating another dominant ‘regime of truth’ with its own limiting capacities? These reflexive questions remain in play as we try new things. If this shift could be shown to reduce stigma and discrimination within mental health care environments, including academia, that would be one important indicator of its value. We propose this practice not as a universal rule, but as one tool among many for redistributing epistemic authority, thus supporting epistemic justice. The mechanisms of power which reproduce authorship are identified by Foucault; however, he does not indicate what the basis of our obligation to act differently would be. This is to be found in a tradition which situates ethical responsibility before ontology.

### 1.7 Ethical Responsibility: Levinas, Derrida and Critchley

The concept of ‘the Other’ has a lengthy philosophical background. Hegel viewed awareness of the self as emerging from contact with others; de Beauvoir [18], demonstrated that societies create categories of people (such as colonised peoples; women etc.) as inferior Others, thereby excluding those categorised as such from being granted full status. This has implications for authorship within co‐productive research; LE collaborators have historically been portrayed as subjects of investigation, not as authors of knowledge.

Levinas radicalised this tradition by reversing its direction. Rather than treating the encounter with the Other as a struggle for recognition, he argued that the first ethical act arises in the face‐to‐face proximity of the Other: when we are presented with the humanity of another, we are called to respond in responsibility, even to the detriment of ourselves. ‘The proximity of a face’ is a primal responsibility, and ‘the other's right to live’ takes precedence over my own [19]. Levinas describes the asymmetry inherent in ethical relationships, arguing that when we encounter another person face‐to‐face, this meeting creates an unbalanced dynamic where their existence and needs take precedence over our own self‐interest [20]. This asymmetry offers a philosophical rationale for listing LE collaborators first, even when they hold less academic status or contribute less to writing or analysis. In a sense, it repositions credit from a quantitative measure of work to a qualitative measure of meaning and responsibility.

‘Othering’ from a critical theory perspective describes the practice of dominant groups denying marginalised peoples the opportunity to achieve full personhood. ‘Othering’, as described by Derrida represents a completely different concept. Even when we have ethical interactions with others, they will be influenced by the underlying structure of power; the intention behind our actions does not preclude the need to challenge the system [21]. Both authors present important concepts related to recognising an obligation to the Other (Levinas), and the obligation being carried out through structures that should be changed (Derrida). Both authors' theories will be relevant to changing how authors are treated and compensated in the future.

Critchley [22, 23] suggests that the two impulses (the critical and the ethical) mentioned above need each other. If ethics does not involve a critique of the underlying system, then it is naive; if critique does not include an ethical commitment, then it is paralysed. When applied to co‐productive research, centring LE in authorship is both an ethical response (recognising the contributions made by collaborators) and a political response (disrupting who is recognised as a knowledge producer). Our own experiences bear this out: Patte was told that openness about LE made them unsuitable for their profession; Stefan found that the structures they worked within had already decided whose knowledge counted. Critchley makes similar arguments that one's ethical obligations cannot be fulfilled without critique. Therefore, LE first authorship is not simply something you check off on your list, but rather a continuous ethical commitment that requires researchers to reaffirm it with each new research project and each time researchers make this decision regarding publication.

### 1.8 Towards Porous Solidarity

Applying Levinas's concept of ‘the Other’ to our work as academics with lived experience presents a productive tension. His framework depends on alterity, radical separation between self and Other, yet our research is grounded in political solidarity and the principle of ‘nothing about us without us’. This appears contradictory: how can we be both ‘us’ (united in collective identity) and attend to ‘the Other’ (preserving difference and alterity)?

**Figure 1:**
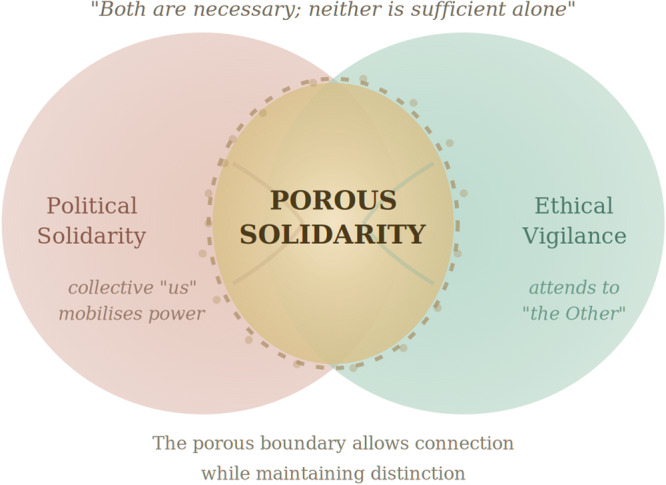
Porous solidarity: A conceptual model linking co‐production, power and ethics.

We propose that these tensions serve different but complementary purposes, requiring what we call porous solidarity (Figure 1). Political solidarity demands ‘us’, a collective identity strong enough to challenge systemic oppression and centre marginalised knowledge. Ethical practice demands attention to difference, ensuring we never presume to speak for others even as we speak with them. The political ‘us’ mobilises collective power; the ethical vigilance preserves individual voices and experiences. Both are necessary; neither is sufficient alone. The ‘interstitial distance’ [22] is a perfect way to capture that; as much as democratic practice requires a productive distance between the citizens and the state, it will require an ethical distance among the members of a research team—so that they can be politically united but still maintain each member's irreducible difference.

In practice, porous solidarity might operate as follows: when our team discussed authorship for this paper, we explicitly named the tension, one author's current institutional position versus the co‐author's experiential authority. Rather than defaulting to convention or imposing a rigid rule, we asked: whose voice does this paper centre? Whose perspective should readers encounter first? This conversation, uncomfortable yet generative, exemplifies porous solidarity, political unity in our shared commitment to centring lived experience, and ethical vigilance about our different positions within academic hierarchies. In our different contexts, we each have the moral authority to speak.

The inevitable conflicts arising from the complex positionality of each one of us, brings these issues into focus every time researchers from different parts of society determine which voice gets to lead. The transparent positionality that we have provided as co‐authors of this manuscript is itself an act of porous solidarity. We argue that the LE collaborator in co‐production research should be listed first because, ethically, their lived experience and stake in the issues are primary: it is their life and community that will ultimately benefit from and be impacted by the research. As researchers, in Levinas's view, we can think of ourselves as responding to the call of the Other, while simultaneously recognising that within political solidarity, the ‘Other’ is also ‘us’. This dual recognition, political unity and ethical difference is the heart of porous solidarity. Unity in diversity.

### 1.9 Addressing Concerns

Several concerns commonly arise when considering LE‐first authorship. One concern relates to tokenism, the perception that an LE collaborator is placed in the first‐author position without having contributed substantially. However, if co‐design is done well, all LE partners should be integrally involved [24, 25]. If they help shape the project from the start and determine what data to collect and how to interpret it, conceptually, they have contributed at least as much as an old‐school first author, and in some respects more. In our team, everyone is involved throughout, including revisions. In this way, the resulting text centres the LE perspective as the primary voice, rather than treating it as something appended through quotations.

The structural barriers that exist in academic publishing can make it even more difficult to navigate for those contributors who have no experience with scholarly publication and those who are entering research via a lived experience role. There is arguably potential for a more inclusive model of scholarship in which research teams publish together under one group name. However, at present, there is no clear way for groups to write collaboratively that escapes the politics of individual authorship. At present, group bylines are frequently accompanied by named individual authors and, like all forms of group authorship, rely on identifiable individuals for both credit and accountability [26] and will contribute to be used for indexing and career advancement purposes. When a link between identified individuals and a piece of research is absent, then responsibility can become diffuse, contributors to a particular paper can become ambiguous and the visibility of each contributor's input into a paper can be diminished [26]. Therefore, group authorship may blur the visible hierarchy of authorship but will not necessarily dismantle the individualised structures of reward upon which academic careers continue to rely.

In co‐produced research, our primary concern is that the collaborative nature of the research can be recognised, and that the voice and perspective centred by the research remains visible in the academic record. While a collective byline can recognise the teamwork involved in producing the research, it can also obscure whose lived experience is the basis of the analysis or whose perspective is first encountered by the reader. Instead, the approach we propose, LE‐first authorship, might be a more practical approach to addressing the issue of redistribution of epistemic visibility towards the person whose lived experience has the greatest influence over the research.

Another consideration is whether a nonacademic LE first author understands what first authorship entails and feels comfortable consenting to a public role. Teams should have an open discussion about preferences and comfort levels. The need for such discussions exists for nonacademic contributors to the research, as well as for researchers with lived experiences whose identities may be complex, partially disclosed or intentionally undisclosed. If a person prefers not to raise their profile as first author, this does not prevent them from being acknowledged in other high‐profile ways (e.g., as a co‐corresponding author). The goal here is not to change authorship order for its own sake, but to remove the default assumption that an institutionally positioned researcher must lead the paper. Instead, the default should be to lead with the voice that the research intends to amplify.

We suggest teams include explicit language in Author Contributions statements to make these choices transparent, such as: ‘[Name] contributed lived‐experience expertise that substantially informed study conception and data interpretation; [Name] is listed as first author to reflect this foundational contribution to the work’. This approach could equally embrace the valued LE of academically qualified members of the team too. More broadly, the proposed approach for first authorship by LE is aligned with community‐led research practices where affected communities do not just define research questions and priorities [27] but also decide who gets credit for their contribution [28, 29].

## 2 Conclusion

Authorship may appear to be a trivial aspect of the larger research enterprise, but as discussed in this manuscript, it represents a microcosm of power and voice in the production of knowledge and in addressing epistemic injustice. This is particularly the case in co‐production research, where we seek to challenge hierarchies that silence voices and to value the contributions of actors and stakeholders. By centring lived experience collaborators in publications, and by openly acknowledging our own LE contribution without shame, we can disrupt authorship hierarchy and the disconnect that often reinforces existing power relations.

These shifts are more than symbolic. First author placement of experience‐based experts where appropriate, plus explicit acknowledgement of all researchers' relevant LE where freely offered, and porous solidarity in relation to institutional and journal policymaking could help transform how research impacts are assessed. Funding bodies and universities may create participatory authorship assessment and evaluation processes as part of promotion and grant evaluation. Journals may develop authorship guidelines for describing lived‐experience leadership.

This reflection does not intend to provide a solution, but to open a conversation. What possibilities will arise for other research teams interrogating these questions? We acknowledge that this manuscript itself participates in some of the contradictions it examines. We write about co‐authorship while navigating constraints on how we can name our own positionalities. We use academic forms while critiquing them. Yet these tensions are not failures, they reveal the limits of what individual choices can accomplish within existing structures. Each decision to prioritise LE in authorship contributes to broader cultural change. While no single paper can dismantle epistemic injustice or promote epistemic justice, collective practices can shift discourse about whose knowledge matters and how we recognise it.

Our focus has been on health‐related co‐production; however authorship structures in which we operate today are products of much larger historical contexts than can be addressed by one paper (including e.g., colonialism; racial hierarchies; and gendered intellectual labour). Critical Race Theory and Settler Colonial Scholarship demonstrates that the constructs of ‘knowledge’ and ‘expertise’ upon which academic publishing operates were developed as part of systems designed to limit and/or eliminate Indigenous and other marginalised forms of knowledge. There may be opportunities for future research to explore how these deeper structural elements of our academic system affect authorship across disciplines and to develop community‐based governance of editorial practice.

We conclude with a call to action. We invite co‐design researchers to test LE‐first authorship practices in their own research teams, and to report on the impact on both the quality of the research produced, team functioning and community engagement. We also invite journal editors to develop authorship guidelines that explicitly address the roles of LE contributors and that provide models for transparency in attribution. Finally, we invite funding agencies to review their evaluation criteria to determine whether they either support or disrupt the hierarchies we have identified in this paper. This paper is simply one small contribution to what we believe will need to be an ongoing collective dialogue.

## Author Contributions

**Patte Randal:** conceptualization, writing – original draft, writing – review and editing, supervision. Patte contributed lived‐experience expertise that substantially informed study conception and data interpretation; Patte is listed as first author to reflect this foundational contribution to the work. **Stefan Heinz:** conceptualisation, writing – original draft, writing – review and editing, supervision, visualisation.

## Funding

The authors have nothing to report.

## Conflicts of Interest

The authors declare no conflicts of interest.

## Data Availability

The authors have nothing to report.
